# Study on Low-Velocity Impact Damage and Residual Strength of Reinforced Composite Skin Structure

**DOI:** 10.3390/ma13112573

**Published:** 2020-06-05

**Authors:** Hanhua Li, Qiuhua Zhang, Jiale Jia, Chunming Ji, Bing Wang, Shi Yan

**Affiliations:** 1 Department of Astronautic Science and Mechanics, Harbin Institute of Technology, Harbin 150001, China; 13B918023@hit.edu.cn (H.L.); siae_123@126.com (Q.Z.); 2 Beijing Institute of Astronautical Systems Engineering, Beijing 100076, China; 3 Department of Engineering Mechanics, Harbin University of Science and Technology, Harbin 150080, China; 15122237012@126.com; 4 National Key Laboratory of Science and Technology for Advanced Composites in Special Environments, Harbin Institute of Technology, Harbin 150080, China; jichunmingjoel@163.com

**Keywords:** reinforced composite plate, low-velocity impact, compression after impact

## Abstract

In order to better understand the damage tolerance of reinforced composite plates, the impact damage of the reinforced composite plates was investigated under low-velocity impact test. The experimental results show that the impact of different positions and energies causes different degrees of damage to the specimens, including but not limited to ply fracture, internal delamination of the skin, and debonding of the stiffeners and skin. After impacting, the specimens were tested in an axial compression. The results show that the ultimate bearing capacity of the specimen is also affected by different forms of impact. The impact point has the greatest influence on the specimen while it locates at the intersection of longitudinal and transverse bars. Compared with the intact specimen, the ultimate load carrying capacity was reduced by 16.83% and 44.02%, while the specimen impacted by 15 J and 30 J, respectively. The compression failure mode of the damaged specimen is mainly the breakage of the stiffeners and the delamination of the skin.

## 1. Introduction

Carbon fiber-reinforced composites (CFRP) have been widely used in the aerospace industry due to their high specific strength and stiffness, performance design, corrosion resistance, and overall shape [1,2,3,4,5,6]. The stiffened composite structure can reduce the weight of structure due to its own material and structural characteristics, which has a great impact on the weight reduction of modern aircraft. Therefore, the research on the stiffened composite structure has special significance in modern times. However, the reinforced composite skin structure as a kind of composite material has its shortcomings that cannot be ignored, that is, the impact resistance is weak [7,8,9,10]. When the reinforced composite skin structure is formed, processed, transported, operated, or repaired, if it is impacted by foreign objects, it may form invisible defects and damages in the structure, including matrix cracking, interface delamination, and fiber breakage. These damages undermine the integrity of the structure, resulting in a significant reduction in the carrying capacity of the structure during service. In order to ensure the safety and reliability of the structure, it is necessary to control the expansion of the defect within a certain range under the action of the load, so that the residual bearing capacity of the structure can meet the requirements [11,12,13,14]. Therefore, the study of residual strength after impact of stiffened composite structure is particularly important.

In recent years, the researchers have paid more attention on the buckling behavior of reinforced composite skin structure. Yazdani et al. [15] has experimentally studied the buckling behavior of thin-walled stiffened cylindrical shells of different types of stiffeners, and obtained that increasing the number of spiral stiffeners under the axial load is more efficient to change the grid type. Through the axial compression test of the advanced grid-reinforced composite cylindrical shell, the initial buckling and post-buckling response of the specimen during loading were studied, and the optimal design of the cylindrical shell of the concave mold composite is carried out by using the finite element simulation experiment process [16,17]. Liu et al. [18] has investigated the compressive buckling behavior of stiffened composite panels, the results showed that the local buckling increases with the loading until the ribs are unstable.

In addition, the bearing capacity of the specimen after impact has also been investigated by the researchers. Ostré et al. [19] has performed low-energy impact and compression test after impact on CFRP structures to establish the edge impact damage scenario. The results showed that the damage types developed from the edge impact and the skin impact are similar. However, the compressive fiber failure becomes crucial in the case of edge damage. Greenhalgh et al. [20,21,22] conducted a large number of low-velocity impact experiments to explore the effect of damages on the failure mechanisms of the stiffened composite plate, and the conclusions presented that the closer the distance between the impact point and the stiffeners, the smaller the damaged area in the skin, which were different with the ones documented in other studies [23]. The difference might relate to the specimen configurations. Bogenfeld et al. [24] has presented an analytic scaling approach to analyze structural impact scenarios on a small reference coupon. It was found that a single reference coupon is enough to assess large areas of structure regarding low-velocity impact. Tan et al. [25] has studied the effects of impact damage on the compression properties of single T-reinforced plates. It was found that the impact damage reduces both the buckling load and the failure load.

Currently, many scholars have paid more attention on the impact experiments of stiffened composite structures. However, the choice of impact position was relatively simple, focusing only on the center of composite skin [26,27]. In addition, there is less research on the residual compressive strength after impact of reinforced composite skin structure [28,29]. In this case, the impact experiments using different impact positions and energies were performed on the reinforced composite panels, which have used the rectangular single-sided skin composite panel that is widely used in the rocket stage. The impact responses of the specimens were analyzed to predict the damage. Moreover, the compression tests after impact were conducted on the damaged specimens, the compression properties and failure modes of the specimens were discussed.

## 2. Materials and Methods

### 2.1. Experimental Materials

**Figure 1:**
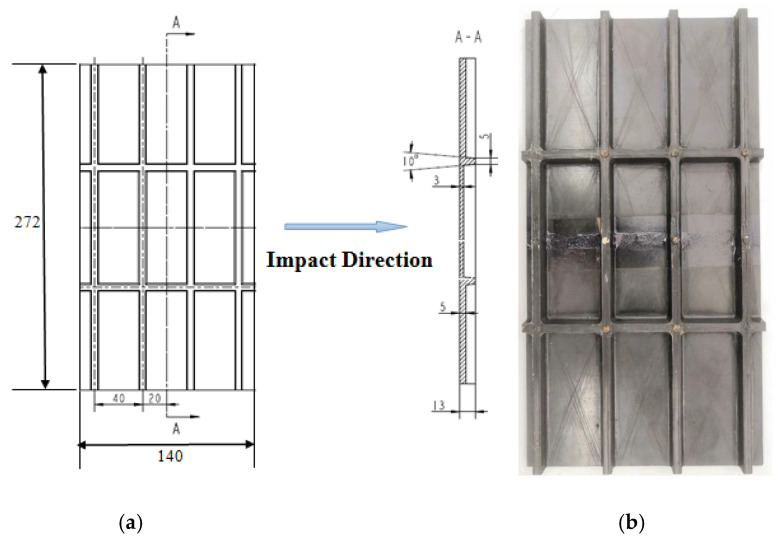
(**a**) The geometry dimension (scale in mm). (**b**) The reinforced composite skin structure specimen.

Fiber-reinforced composites consisting of skins and stiffeners were used in this study, as shown in Figure 1. The structure of the specimen is designed according to the parameters of the hood, the interstage section and the tail section of triangular grid structure. The length and width of the specimen are 272 and 140 mm, respectively. The skin has a thickness of 3 mm. All stiffeners and skins co-bonded in the mold. During the compression process, the edge of the skin should be thickened by 2 mm of tabs to prevent the specimen from instability as presented in Figure 1. In addition, the height of the specimen should be kept within a reasonable range. In order to eliminate the boundary effect of the specimen, it is necessary to leave a certain width of the specimen. The height and width of the stiffeners are 10 mm and 5 mm, respectively, and the stiffeners are provided with a certain angle along the height direction, mainly to improve the integrity of the specimen. The reinforced fiber of the specimen is MT300, and the resin matrix is 603 epoxy resin prepreg. Its mechanical properties were tested by unidirectional composite laminates as shown in Table 1. The lay-up of skin is [±45°/0°_2_/45°/0°_2_/90°/−45°/0°]_s_.

### 2.2. Experimental Methods

#### 2.2.1. Low-Velocity Impact Test

**Figure 2:**
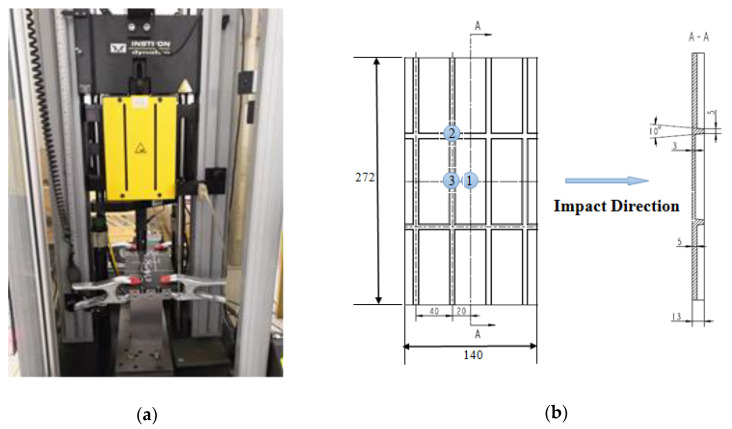
(**a**) Experimental equipment for the drop-weight impact tests. (**b**) The impact positions (scale in mm).

The drop-weight impact testing machine of Instron 9250 HV (Instron, Boston, MA, USA) was adopted to carry out the impact test, as shown in Figure 2a. The fixing device which was special design and the pneumatic clamp are used to fix the specimens. When the drop hammer rebounds, the pneumatic valve pops up to prevent the secondary impact of the drop hammer. The total mass of the drop hammer is constant at 8.765 kg for all impact tests. The impactor is a rigid hemisphere with a diameter of 12.7 mm.

In this research, the low-velocity impact position is as depicted in Figure 2b. These points are named 1, 2, and 3. The parameters of the experiments with different impact positions and energies are shown in Table 2. Position 1 is located on the center of the skin surface; Position 2 is the corresponding point of the midpoint of a single stiffener; Position 3 is the corresponding point to the intersection of the two stiffeners. Before the formal impact tests, a trail impact test with energy of 30 J was first carried out. It was found that the specimen was penetrated in Position 1, and the separation of ribs and skin panel occurred obviously at positions 2 and 3. In order to compare and analyze the damage variation of the stiffened composite structure to different impact energies, energies of 15 J and 30 J were used, which results in varying degrees of visible damage and invisible damage. Under each position and impact energy, which are categorized #2 to #7, three specimens were tested. Moreover, the impact damage was measured by ultrasonic C-scan after the impact test.

#### 2.2.2. Compression Test after Impact

**Figure 3:**
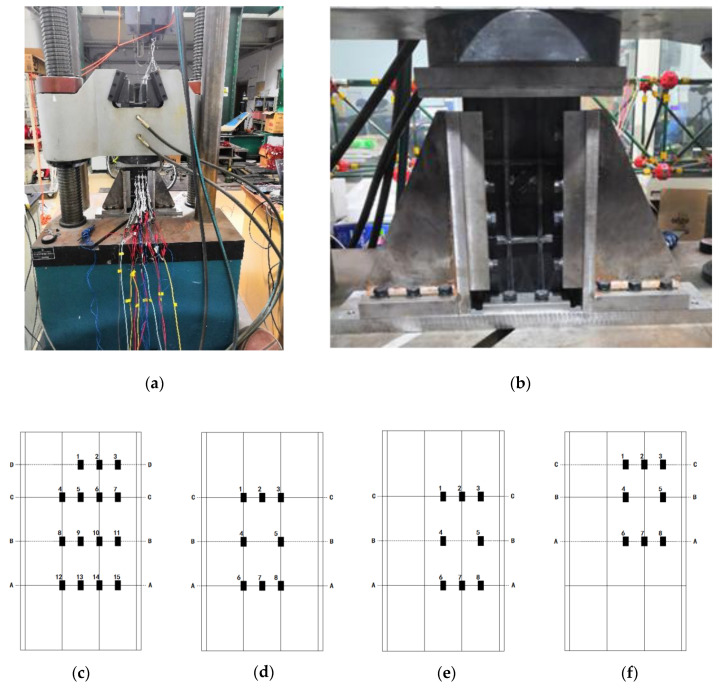
(**a**) The universal testing machine. (**b**) The experimental fixture. (**c**–**f**) The diagram of strain gauge distribution (#1 specimens follow (**c**) distribution, #2/#3 specimens follow (**d**) distribution, #4/#5 specimens follow (**e**) distribution and #6/#7 specimens follow (**f**) distribution.

Compression test after impact (CAI) is one of the commonly used methods to evaluate the effect of impact damage on the structure, which is used to measure the residual compressive strength of the specimens. In this study, compression tests were performed on three intact specimens to compare with CAI experiments, which named as #1. The compression test was carried out on a WAW-1000B hydraulic test machine (Sinter, Changchun, China) with a maximum load of 1000 kN, as presented in Figure 3a. The machine is equipped with two steel platforms. The top platform is fixed and the bottom can be moved under displacement or force control. For a better stability, the force control was applied with an experimental loading rate of 0.5 kN/s. Both sides of the specimen are fixed by clamps to prevent buckling damage, as detailed in Figure 3b. During the experiment, the strain gauges are used to monitor the strain variation of the specimens. In order to better characterize the strain variation around impact damage under compression, the distribution of the strain gauges is illustrated in Figure 3c–f. All the strain gauges were placed on the outer skin surface. (#1 specimens follow (c) distribution, #2/#3 specimens follow (d) distribution, #4/#5 specimens follow (e) distribution and #6/#7 specimens follow (f) distribution.)

## 3. Results

### 3.1. Impact Response

**Figure 4:**
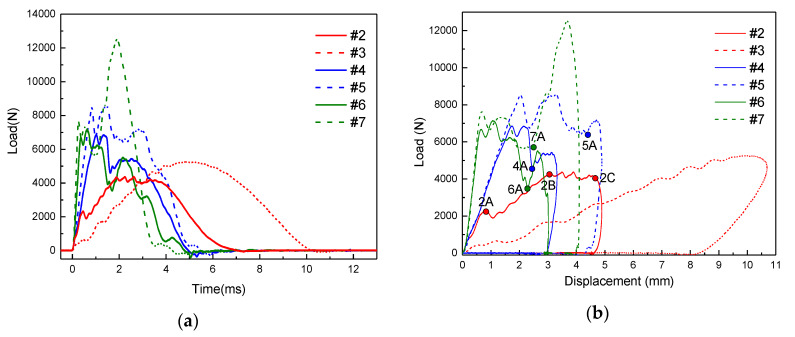
The curves of (**a**) load–time and (**b**) load–displacement for the impacts on the stiffened panels (15J: solid lines; 30J: dashed lines).

The curves of load–time and load–displacement are shown in Figure 4. For the #2 specimen, the impact process is mainly divided into four stages. In the first stage, the impact force increases linearly with time, which rises rapidly to point 2A (the first marked point of #2 specimen) as presented in Figure 4b. At this stage, the specimen is elastically deformed, indicating that there is no damage. It can be seen that the bending rigidity of the material does not change. When the load reaches point A, it drops rapidly to 1800 N, which is caused by the delamination and unstable expansion of the specimen. In the second stage, the slope of the load–displacement curve and the bending rigidity of the specimen decreased, indicating that the specimen had been destroyed. From point 2B to point 2C, the fiber is seriously damaged and the impact force reaches the peak value. After point 2C, the impact force drops rapidly. Moreover, the load–time curve of #3 specimen is roughly symmetrical. When the load reaches 700 N, it decreases for the first time and the specimen is damaged. Then, with increasing load, the specimen continues to be damaged. Due to the high energy, the specimen is destroyed by the heavy hammer. However, there is a discrepancy between the load-displacement curves of #2 and #3 specimens which suggests a different indentation stiffness. The difference is probably related to the impact of #3 specimen that not placed as intended. Furthermore, the impact points of #4, #5, #6, and #7 specimens are in the upper part of the stiffener. Due to the support of the stiffeners, the impact load continues to rise after reaching point 4A for #4 specimen, point 5A for #5 specimen, point 6A for #6 specimen, and point 7A for #7 specimen. Compared with #4 and #5 specimens, due to the lower impact position of the #6 and #7 specimens is supported by the stiffeners in both the longitudinal and transverse directions, the impact loads increase greatly after passing the points 6A and 7A separately, and then the impact force is continuously reduced as the impactor rebounds.

### 3.2. External Damage

**Figure 5:**
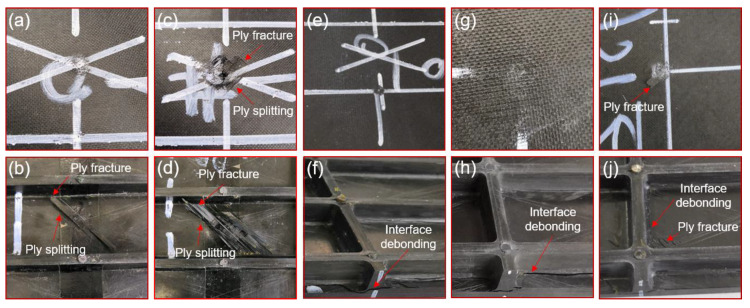
External impact damage of panels: (**a**,**b**) Out of plane damage of #2 specimen; (**c**,**d**) Out of plane damage of #3 specimen; (**e**,**f**) Out of plane damage of #5 specimen; (**g**,**h**) Out of plane damage of #6 specimen; (**I**,**j**) Out of plane damage of #7 specimen.

Low-velocity impact damage is formed on the surface of the specimen. However, due to the different impact locations and impact energies, the forms of external damage are also different. As shown in Figure 5a,b, there is out of plane damage on the #2 specimen. The hammer forms a pit of about 3 mm in diameter on the upper surface of the specimen. The damage of the lower surface is obvious. The matrix cracks are along the direction of laying layer, and the cracks are limited by the stiffeners, with a length of about 47 mm. The damage of the #3 specimen is depicted in Figure 5c,d. The diameter of the pit on the upper surface is about 8 mm, the ply fracture along the direction of the pavement and the delamination appears at the edge of the pit, the damage on the lower surface of the specimen includes ply fracture and fiber breakage. The crack of the matrix is along the fiber direction. The fracture line formed by the fiber breakage exhibits two states, one is along the 0-degree direction and the other is perpendicular to the fiber direction. This phenomenon is due to the large tensile stress on the back of the specimen during the impact process, which is decomposed into the short axis direction and the fiber direction. The experimental results of the impact point at the lower part without the stiffeners are similar to those of the impact on the laminates. Because of the support of the stiffeners, the impact energy did not reach the damage threshold of the specimen, and there is no obvious damage on the specimen surface. The damage of the #5 specimen is shown in Figure 5e,f. The impact formed a pit with a diameter of about 1 mm on the upper surface of the specimen. In addition, the stiffener/the skin debonding could be found on the lower surface of the specimen. Figure 5g,h present the out-of-plane damage of the #6 specimen. There is no obvious damage on the upper surface, but the lower surface skin is debond from the stiffener. As for the #7 specimen, it has a pit with a diameter of 3 mm on the upper surface as shown in Figure 5i, and the plate layer at the edge of the pit cracks along 45°. Same with the #5 and #6 specimens, the impact induces the lower surface to delamination and the stiffener/skin debonding as presented in Figure 5j.

### 3.3. Internal Damage

**Figure 6:**
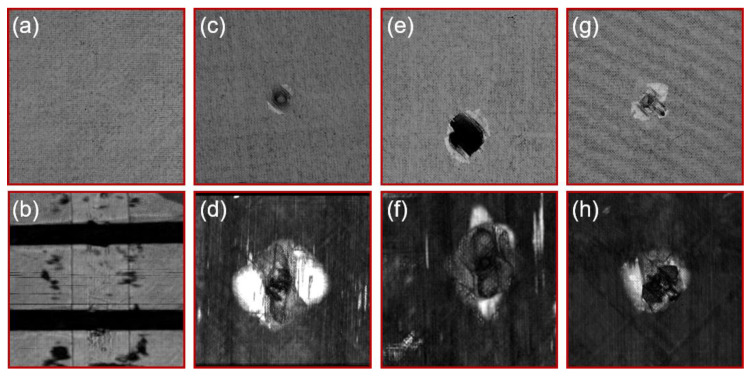
Internal impact damage of panels: (**a**,**b**) Specimen without damage before impact; (**c**,**d**) Internal damage of #2 specimen; (**e**,**f**) Internal damage of #5 specimen; (**g**,**h**) Internal damage of #7 specimen.

The internal damage of the specimen was detected by an ultrasonic C-scan system. Figure 6a,b shows the C-scan of the specimen before impact, and there are no defects and damages inside the specimen. Figure 6c shows the impact pit on the upper surface of the #2 specimen, and Figure 6d presents the internal damage of the intermediate layer of the #2 specimen. In addition, the impact damage areas of #2, #5, and #7 specimens from C-scan are illustrated in Table 3. Interestingly, the shape of the damaged areas looks peanut-shaped with the major axis in the longitudinal direction, which was also observed by Wiggenraad and Feng [23,30]. Compared with Figure 5c,d, the interior damage is much serious than the external damages. Moreover, the greater the impact energy is, the greater the internal damage is. Due to the small impact energy and the support of the lower stiffener, no obvious damage was observed in the #4 specimen. The internal damage of the #5 specimen is shown in Figure 6e,f. As the impact point is above the stiffener, the stiffener absorbs part of the energy during the impact. Therefore, the damage in the skin is much less than the damage at position 1. Figure 6g,h show the internal damage diagram of the #7 specimens. It can be seen that the internal damage is approximately elliptical. The damage area is smaller than the damage at position 1.

## 4. Discussions

### 4.1. Analysis of Load–Displacement Curve and Strain

**Figure 7:**
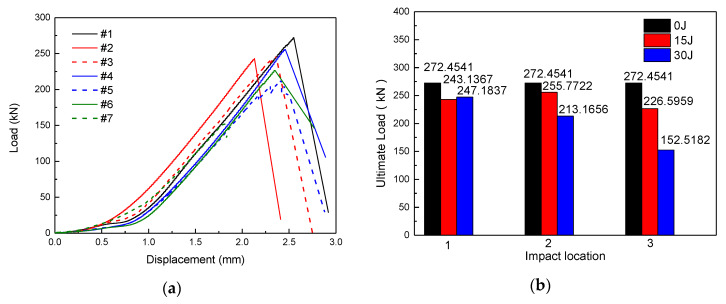
(**a**) The load–displacement curves of the specimens. (**b**) Ultimate load histogram.

The load–displacement curves of the specimens are shown in Figure 7a, which present a similar trend. Therefore, #1 specimen is taken as an example for illustration. In the initial stage of compression, the curve is flat because of the gap between the specimen and fixture. After the specimen is in full contact with the fixture, the slope of the curve remains constant. When the ultimate load is reached, the specimen is completely destroyed and the load is reduced. The load–displacement curve of the #5 specimen has a zig-zag shape before reaching the peak value because the specimen is partially destroyed during the compression process, and the previous balance system disappears. As the ultimate load carrying capacity of the specimen has not been reached, the specimen reaches a new balance after unloading. This process is repeated until the ultimate load carrying capacity of the specimen is reached, then the specimen is crushed. As shown in Figure 7b, compared with the intact specimen, the load bearing capacity of the specimens after the impact were reduced, but the degree of decline was different. The ultimate load carrying capacity of #2 and #3 specimens were 243.1367 kN and 247.1837 kN, and the bearing capacity decreased by 10.76% and 9.27%, respectively. The impact damage has little effect on the bearing capacity of the specimens. Since there is no stiffener at the impact point, the damage caused by the impact only exists in the skin, and the stiffener is not affected. Moreover, the load carrying capacity of #4 and #5 specimens decreased by 6.12% and 21.76%, respectively. Due to the presence of the stiffeners, the impact energy of 15 J did not exceed the damage threshold of the specimen, and then the bearing capacity of the specimen decreased little. However, while the impact energy is increased to 30 J, the stiffeners and the skin are debound in a large area due to the serious impact damage of the specimen (Figure 5f), so the bearing capacity of the specimen is reduced. Furthermore, the load carrying capacity of #6 and #7 specimens decreased by 16.83% and 44.02%, respectively. As the impact position is above the intersection of the longitudinal stiffeners and the transverse stiffeners, the impact causes a large area of stiffeners/skin debonding (Figure 5h,j), which seriously affects the bearing capacity of the structure, and greatly reduces the bearing capacity. Obviously, compared with positions 1 and 2, the impact damage at position 3 has a greater negative effect on the bearing capacity of the stiffened composite plates.

**Figure 8:**
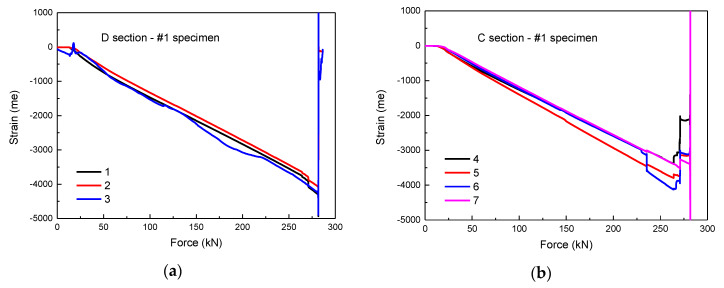

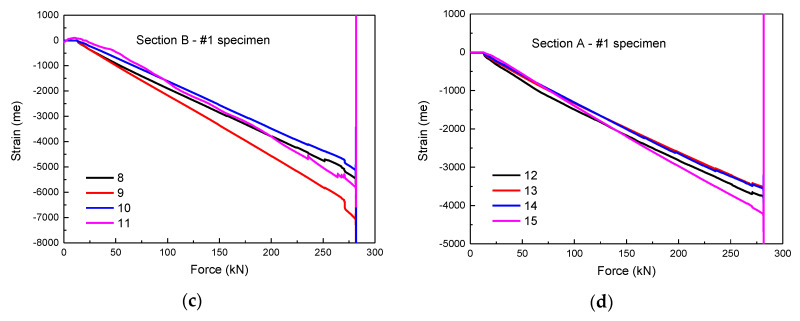
The strain-force curves of the #1 specimen: (**a**) local strain of section D (Points 1–3); (**b**) local strain of section C (Points 4–7); (**c**) local strain of section B (Points 8–11); (**d**) local strain of section A (Points 12–15).

In order to analyze the failure mode of the specimen, strain gauges were attached at different positions of the specimen to obtain the strain at each position. The position of strain gauges of the intact specimen is shown in Figure 3a. As presented in Figure 8a–d, the strain–force curves of the intact specimen are observed, which shows the negative strain values at each position of the specimen. Therefore, the specimen is subjected to compression deformation without the tendency of buckling failure. The specimen was crushed while the force was 281.7 kN. During the compression process, the curve shows a good linearity. Comparing the strain values of each horizontal section, the maximum strain of the section B (Points 8–11) is ~7000 × 10^−6^, and the strain of other sections is less than 4500 × 10^−6^, so the compression failure is generated from the section B for intact specimen.

**Figure 9:**
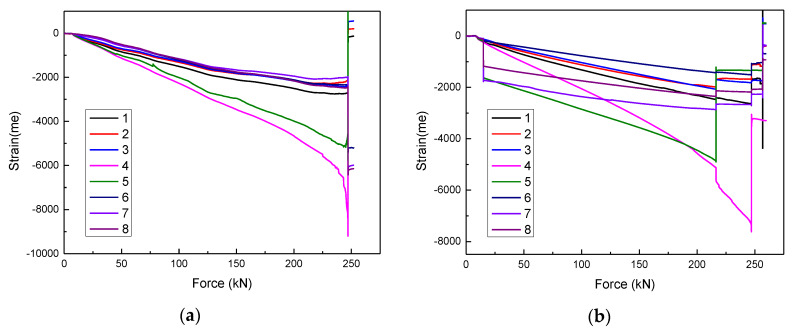

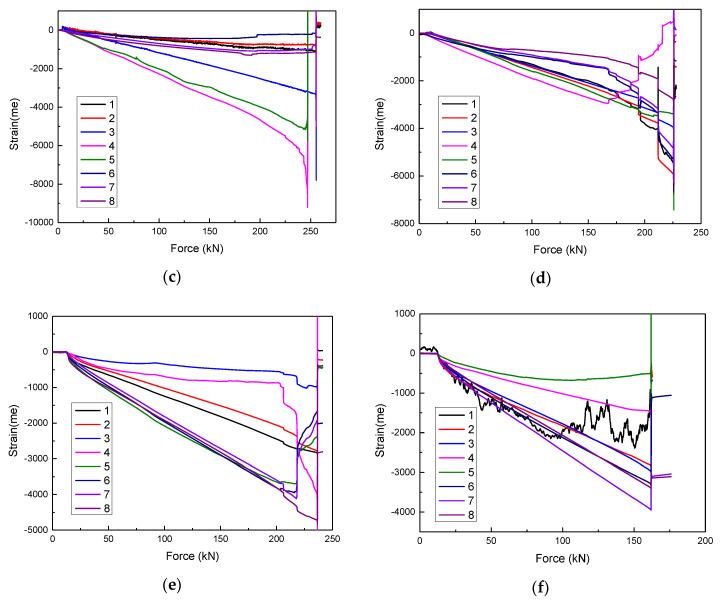
The strain–force curves of the specimens: (**a**) #2 specimen; (**b**) #3 specimen; (**c**) #4 specimen; (**d**) #5 specimen; (**e**) #6 specimen; (**f**) #7 specimen.

The strain–force curves of the #2 and #3 specimens are shown in Figure 9a,b; the curve trend of damaged specimen is similar to that of undamaged specimen. All positions of the specimen are compressive strain and keep linear increase, indicating that only compression deformation occurs. Under the same loading mode, the less the loading time, the lower the bearing capacity. The specimen was crushed while the force was ~250 kN, which shows that the impact weakens the specimen. The strain of the section B (Points 4 and 5) is much larger than that of the other positions, which indicates that the section B is the initial position of the compression fracture of the #2 and #3 specimens. Moreover, Figure 9c,d presents the strain-force curves of the #4 and #5 specimens. The increase of strain slope of section A (Points 6–8) and section C (Points 1–3) after 170 kN is due to the debonding of stiffener/skin caused by the impact of position 2. The #5 specimen was crushed while the force was about 225 kN. The skin at the position 4 is arched to cause the strain gauge to rebound and generate tensile stress during the compression process. Therefore, the strain at position 4 of the #5 specimen changes from compressive strain to tensile strain. In addition, the strain curves of the #6 and #7 specimens are presented in Figure 9e,f. The impact of position 3 caused a large area of the stiffener/skin debonding, resulting in a much lower failure force. The #7 specimen was crushed while the force was 162 kN.

### 4.2. Compression Failure Mode and Failure Morphology Analysis

**Figure 10:**
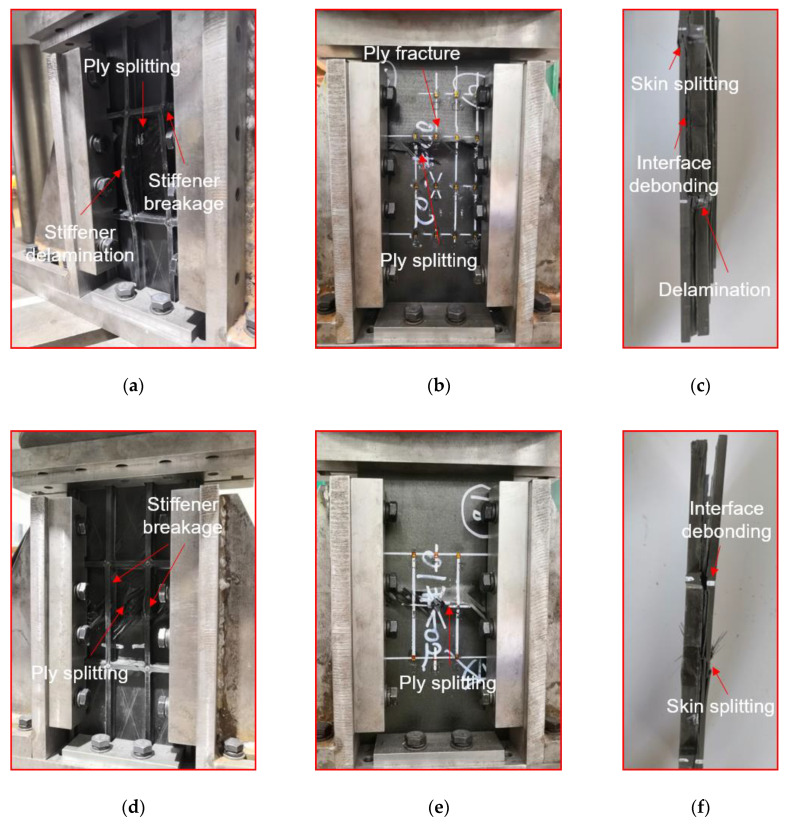

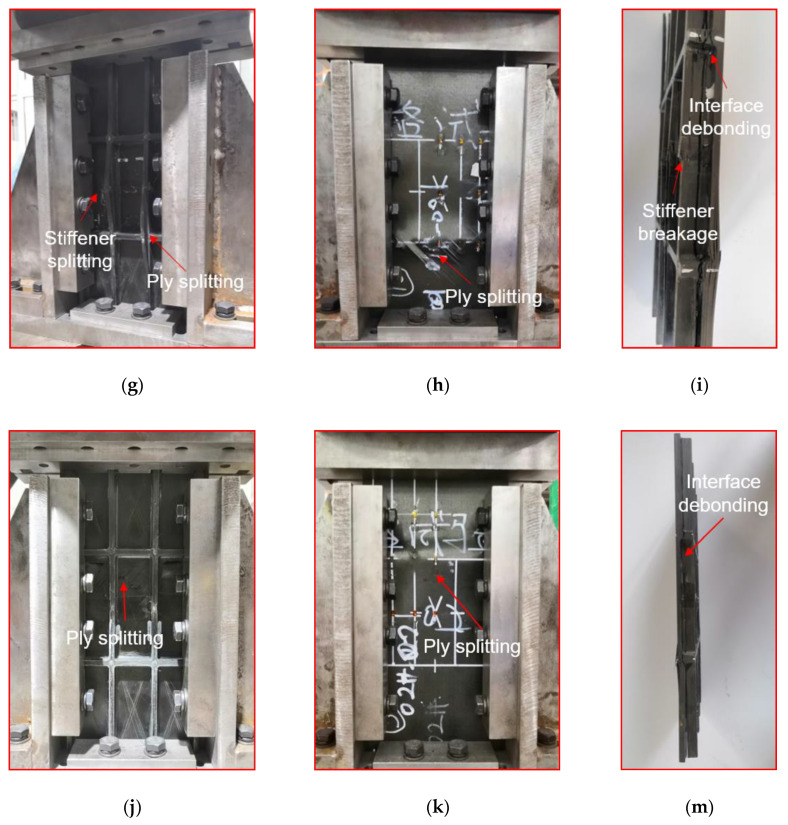
Damage diagram of specimen after compression: (**a**–**c**) compression damage of #1 specimen; (**d**–**f**) compression damage of #3 specimen; (**g**–**i**) compression damage of #4 specimen; (**j**–**m**) compression damage of #7 specimen.

The typical compression failure of the intact specimen (#1) is shown in Figure 10a–c. Before the failure, the specimen was displaced uniformly under the compressive load. With the increase in compression load, the specimen becomes slightly unstable. When instability occurs, part of the load in the skin will be transferred to the bars and the stiffeners. Therefore, the damage tends to initiate in the interface of skin and stiffeners. Then, the damage of interface expands with the compressive load increasing, which triggers the further decrease of carrying capacity of skin. While the load exceeds the ultimate bearing capacity of the specimen, the stiffened plate will collapse, resulting in the delamination of the skin [23]. As presented in Figure 10a, the stiffener is broken caused by compression and the fracture position is randomly distributed. The stiffener fracture led to serious delamination and ply fracture (shown in Figure 10c). Moreover, the stiffener/skin interface is debound along almost the entire spanning length. On the other side of the specimen, the skin is broken along the 45° direction, resulting in serious ply fracture and ply splitting.

The compression failure diagrams of the impact point of Position 1 (#3 specimen) are shown in Figure 10d–f. The failure modes of #2 and #3 specimens are similar, which forms on the side of the stiffener including (shown in Figure 10d): stiffener breakage, stiffener delamination, stiffener and skin interface debonding, and severe delamination of skin. As presented in Figure 10e, on the side of the skin, the intermediate section bearing capacity is weak due to the impact. During the compression process, the specimen is broken along the intermediate section, and the skin is arched outward on the same horizontal line. The skin is bent toward the side of the skin, and the failure modes include the ply fracture and the ply splitting. Moreover, Figure 10g–i shows the compression failure diagram of impact point of Position 2 (#4 specimen). The stiffener fracture of the specimen appears before the critical load. The final failure modes of the specimen are the fracture of the stiffeners and the local delamination of the skin caused by the fracture of the stiffeners. Furthermore, the compression failures of impact point of Position 3 (#7 specimen) are presented in Figure 10j–m. Before reaching at the ultimate load, first the stiffener of the specimen breaks. Due to the fracture of the stiffeners, the bearing capacity of the specimen is insufficient, and then the skin is compressed and layered. The impact point of Position 3 resulted in a large area of debonding between the skin and the stiffeners, leading to a complete separation of the stiffeners and the skin after the compression failure of the specimen. It is a common phenomenon that the damage position of the skin passes through the impact point, which shows that the bearing capacity of this point is very weak after being impacted. Moreover, the impact is causing out of plane deformation of the panel leading to inter-laminar shear failure at stiffener–skin interface. Due to low fracture and mechanical properties of resin, debonding occurs, leading to severe degradation of overall load bearing capacity of the skin panels.

## 5. Conclusions

In this study, the damage forms of the reinforced composite skin structure under different impact energies and the influence of the impact on the compression performance of the specimen were investigated. The conclusions are as follows.

- While the impact point is at the center of the skin surface, most of the impact energy is absorbed by the skin, the damage of the skin is the most severe, and there is no debonding between the skin and the stiffeners. Moreover, when the impact point is located at the corresponding point of the midpoint of a single stiffener and the corresponding point to the intersection of the two stiffeners, part of the impact energy is absorbed by the stiffeners. The damage area of the skin reduced while there exists the debonding between the skin and the stiffeners. In addition, the damage area formed by the impact in the skin is usually “elliptical”.
- Through the compression test of the reinforced composite skin structure after the impact, it is concluded that the impact has an influence on the bearing capacity and failure modes of the reinforced skin structure. The test results show that the bearing capacity of the specimen decreases after impact in a certain range: the bearing capacity of the specimen is negatively correlated with the impact energy. The impact points at the center of the skin surface and the corresponding point of the midpoint of a single stiffener have little effect on the bearing capacity. However, the bearing capacity of the specimens with the impact point of the corresponding point to the intersection of the two stiffeners has a significant decline.
- Compared with intact specimen, the impact does not change the compression failure mode of the specimens, and all types of specimen failure are caused by the stiffener fracture and the skin delamination.

In the compression test, the propagation mode of the internal damage is still unclear. In the next step, a finite element model will be established based on the experimental results to further study the details of damage propagation in CAI.

## Figures and Tables

**Table 1:** Material properties.

Properties		Value
Longitudinal Young’s modulus	*E*_11_	148 GPa
Transverse Young’s modulus	*E*_22_ = *E*_33_	10.5 GPa
Shear modulus	*G*_12_ = *G*_13_	5.6 GPa
	*G*_23_	3.5 GPa
Poisson’s ratio	*𝒱*_12_ *=* *𝒱*_13_	0.31
	*𝒱*_23_	0.2
Longitudinal compressive strength	*Y_c_*	1555 MPa
Transverse compressive strength	*X_c_*	247 MPa
Plane shear strength	*S*	91.5 MPa
Fiber volume fraction	*V_f_*	60%
Ply thickness	*d*	0.125 mm

**Table 2:** Test parameters.

Specimen Number	Impact Energy [J]	Impact Position
#1	/	/
#2	15	1
#3	30	1
#4	15	2
#5	30	2
#6	15	3
#7	30	3

**Table 3:** Impact damage of #2, #5, and #7 specimens from C-scan.

Specimen Number	Scan Depth [mm]	Damage Area [mm^2^]
#2	1.5	342
#5	1.5	197
#7	1.5	93
